# Grain area data and yield characteristics data in rapid yield prediction based on rice panicle imaging

**DOI:** 10.1016/j.dib.2019.104667

**Published:** 2019-10-15

**Authors:** Haonan Zheng, Sanqin Zhao, Yutao Liu

**Affiliations:** College of Engineering/Jiangsu Key Laboratory of Intelligent Agricultural Equipment, Nanjing Agricultural University, Nanjing 210031, China

**Keywords:** Six rice cultivars, Weight parameters, Panicle images, Grain area

## Abstract

To explore the relationship between the attributes of the rice panicle and its weight parameters, 6 different rice cultivars from Sihong City, Jiangsu Province, China were selected for sampling in 2017. Then, their weight parameters were measured. The images of rice panicles were scanned to obtain grain area. The significant correlation between the grain area and the panicle weight was found on the base of the analysis for the data obtained [1]. Now the weight and area data were present here for exploring the rapid yield estimation models and crop phenotype research.

**Table:** Specifications Table

Subject area	*Agricultural and Biological Science*
More specific subject area	*Image processing, crop yield estimation*
Type of data	*Table, figure*
How data was acquired	*Weight parameters were weighed by Electronic balance (Mettler Toledo, PL402-L, Mettler Toledo Group, Switzerland.)* *The images of the panicles were obtained by Scanner (Epson Perfection V370, Seiko Epson, Indonesia)* *The images were processed by Matlab and then the grain areas were obtained.*
Data format	*Raw, analysed, descriptive and statistical data*
Experimental factors	*Rice cultivar, panicle weight parameters, grain area*
Experimental features	*Collect 1 m*^*2*^*of rice panicles, air dry in a room for two weeks, separate the branches so that they do not overlap. Then, scan to obtain images, rice yield trait data and grain area data.*
Data source location	*Sihong Comprehensive Demonstration Base of Modern Agricultural Science and Technology in Huaian City, Jiangsu Province, China (33°29′2.00″N, 119°01′13.51″E)*
Data accessibility	*Data are included in this article and part of the raw data uploaded into the system as supplement.*

**Table:** 

**Value of the Data** • These data describe the weight parameters of 6 different rice cultivars, which include the panicle weight, total grain weight and unfilled grain weight. • The data showed that the panicle area was the main determinant of the grain weight for each rice cultivar [1]. • Considering the strategic roles to promoting the intelligence of agriculture, the data from this study can be used by policy makers and researchers to estimate the rice yields rapidly, especially for the breeding, food regulation and trading sectors.

## 1 Data

The dataset contains raw rice grain weight parameters through the measurement and image processing from 6 rice cultivars and 1200 rice panicles. The rice cultivars were denoted as A, B, C, D, E and F respectively. The data files (in.xlsx format) were uploaded into the system as supplementary.

In the distribution characteristics of the panicle weight from different rice cultivars, the skewness and kurtosis coefficient were both close to 0, which indicated that the panicle weights of each group were normally distributed (Table 1).

**Table 1:** The weight distributions of rice panicles of different rice cultivars.

Rice cultivar	Effective sample number	Skewness coefficient	Kurtosis coefficient	Weight distribution histogram
A	199	−0.34	−0.09	
B	200	−0.24	−0.15	
C	200	−0.20	−0.76	
D	200	0.09	−0.37	
E	200	0.14	−0.43	
F	199	0.09	−0.25	

A variance analysis of the rice yield related indices was performed, and the results are shown in Table 2. The values of the indices were significantly lower for cultivar D than for all of the other cultivars except cultivar C, while the values of cultivar E were significantly higher than those of the other cultivars except cultivar A. These results indicated that there were significant differences in the panicle attributes among the six cultivars.

**Table 2:** Analysis of mean and variance of rice panicle parameters.

Rice cultivar	Area (cm^2^)	Panicle weight (g)	Grain weight (g)	Panicle weight minus unfilled grain weight (g)	Grain number per panicle (grain)	Filled grain number (grain)
A	23.85 ± 5.35d	3.52 ± 0.83cd	3.36 ± 0.79c	3.44 ± 0.82cd	149.73 ± 34.18c	132.92 ± 30.21c
B	19.64 ± 5.23b	3.41 ± 0.93c	3.31 ± 0.90c	3.38 ± 0.92c	136.67 ± 37.51b	126.75 ± 33.73c
C	16.32 ± 4.64a	2.81 ± 0.86b	2.68 ± 0.82b	2.77 ± 0.86b	115.09 ± 34.36a	103.99 ± 31.93a
D	16.01 ± 5.22a	2.58 ± 0.88a	2.48 ± 0.85a	2.53 ± 0.87a	117.65 ± 39.07a	103.71 ± 34.72a
E	22.12 ± 6.03c	3.67 ± 0.99d	3.53 ± 0.96d	3.57 ± 0.96d	151.08 ± 41.82c	132.50 ± 34.57c
F	20.38 ± 5.73b	2.83 ± 0.86b	2.72 ± 0.83b	2.74 ± 0.85b	140.18 ± 36.12b	117.58 ± 32.69b

Note: Values marked by different lowercase letters in the same column are significantly different at the 0.05 level (Duncan's test).

### 1.1 The correlation between the rice panicle weight parameters(y) and the grain area(x) for 6 different cultivars

Table 3 shows that the determinant coefficients of grain area and the different weight parameters were similar to each other within a given cultivar; All the *R*^*2*^ of the prediction models are all above 0.8300, this result indicated that the model could predict the panicle weight parameters from grain area well within a cultivar and that prediction accuracy varied slightly among different cultivars.

**Table 3:** The correlation between rice panicle weight indices and grain area of different rice cultivars.

Indices (Y)		A	B	C	D	E	F
Panicle weight	Formula	0.1456x + 0.0487	0.1759x – 0.0337	0.1818x − 0.1482	0.1621x – 0.0247	0.1530x + 0.2836	0.1444x – 0.1170
	R^2^	0.8821	0.9669	0.9576	0.9416	0.8926	0.9244
Panicle weight minus unfilled grain weight	Formula	0.1417x + 0.0613	0.1735x – 0.0198	0.1797x – 0.1504	0.1591x – 0.0265	0.1451x + 0.3580	0.1411x – 0.1386
	R^2^	0.8483	0.9621	0.9487	0.9319	0.8512	0.9102
Filled grain weight	Formula	0.1333x + 0.0913	0.1675x + 0.0000	0.1715x − 0.1419	0.1517x – 0.0157	0.1402x + 0.3187	0.1351x – 0.1279
	R^2^	0.8349	0.9607	0.9460	0.9303	0.8359	0.9049
Total grain weight	Formula	0.1372x + 0.0786	0.1699x − 0.0139	0.1736x − 0.1398	0.1547x – 0.0139	0.1480x + 0.2442	0.1383x – 0.1063
	R^2^	0.8713	0.9657	0.9555	0.9406	0.8801	0.9200

## 2 Experimental design, materials and methods

The methods for obtaining weight parameters and grain area were the same as the paper indicated [1], Panicles of 6 japonica rice cultivars and 200 panicles of each cultivars were sampled, and then the data related to weight were measured with an electronic balance. The panicle images were acquired by a scanner and the images were processed by the feature extraction algorithm of grain area developed for MATLAB [1]. The statistical analysis were performed using SPSS Statistics 25.0.

## 3 Conclusion and implications of the study

The conclusion from the data is that grain weights are mainly determined by its panicle grain areas for each rice cultivars. The results indicate that a new and fast rice yield estimation method could be developed for practical use.
